# Next-Generation Cartilage Repair: Clinical Use of Wharton’s Jelly MSCs and the Emerging Role of AI-Assisted Bioprinting

**DOI:** 10.3390/bioengineering13090995

**Published:** 2026-08-27

**Authors:** Bogusław Sadlik, Magdalena Matuszewska, Wojciech Klon, Ewa Stodolak-Zych, Kamila Rawojć

**Affiliations:** 1 Orthopaedy Facility, College of Physiotherapy in Wroclaw, 54-430 Wroclaw, Poland; 2 Go on Clinic, 54-430 Wroclaw, Poland; 3 St. Luke’s Hospital, 43-309 Bielsko-Biała, Poland; 4 Department of Biomaterials and Composites, AGH University of Science and Technology, 30-059 Krakow, Poland; 5 Department of Biocybernetics and Biomedical Engineering, AGH University of Science and Technology, 30-059 Krakow, Poland

**Keywords:** OA, WJ-MSCs, umbilical cord, osteochondral defect, MSCs, regenerative medicine

## Abstract

The treatment of articular cartilage defects remains a significant clinical challenge due to the tissue’s limited intrinsic repair capacity. This paper presents a review of clinical experiences with the use of Wharton’s jelly-derived mesenchymal stem cells (WJ-MSCs) as a novel therapeutic option for cartilage regeneration. WJ-MSCs offer key advantages, including high proliferative potential, strong immunomodulatory properties, and low immunogenicity, making them suitable for allogeneic applications. This review describes a single-step, dry-arthroscopic technique that was employed for the implantation of WJ-MSCs embedded in a scaffold directly into cartilage defects. Clinical follow-up, supported by MRI evaluation, demonstrated favorable outcomes with evidence of defect filling, improved cartilage surface quality, and sustained functional improvement in patients. These results suggest that WJ-MSC-based therapies, delivered through minimally invasive surgical techniques, represent a safe and effective strategy for cartilage repair, with the potential to become an important alternative to current standard treatments. Recent advances in artificial intelligence (AI) and multimodal bioprinting are opening new perspectives for standardizing regenerative therapies. Machine learning models can predict bioink performance, optimize scaffold design, and integrate real-time imaging feedback such as optical coherence tomography and photoacoustic imaging. These approaches allow closed-loop quality control and the creation of digital twins to ensure biomechanical fidelity of constructs. Incorporating AI-assisted bioprinting with Wharton’s jelly MSCs could accelerate the translation of laboratory findings into reproducible, patient-specific cartilage implants. This manuscript is structured as a translational review of WJ-MSC-based cartilage repair, with AI-assisted bioprinting presented as a prospective future manufacturing direction rather than current clinical practice.

## 1. Introduction

Patients in advanced stages of life who maintain relatively high levels of physical activity are generally considered suboptimal candidates for total [TA] or unicompartmental [UKA] knee arthroplasty [1,2]. There is an ongoing clinical need for orthopedic interventions capable of promoting articular cartilage regeneration in patients for whom conventional techniques—such as bone marrow stimulation via microfracture, nanofracture, or spongiolization—have failed to achieve satisfactory outcomes [3,4]. Concurrently, the extent of joint degeneration in these individuals may be insufficient to justify arthroplasty [5], while their functional expectations for high physical activity remain elevated [6].

To address this unmet clinical need, numerous research groups worldwide are investigating advanced strategies for cartilage repair, emphasizing the use of bioengineered scaffolds in combination with young allogeneic stem cells [7]. Among these approaches, the utilization of umbilical cord-derived mesenchymal stem cells (UC-MSCs) has emerged as particularly promising [8], owing to their accessibility and favorable immunological profile. Initial studies indicate that UC-MSCs exhibit low expression of major histocompatibility complex antigens [8], thereby potentially enabling allogeneic transplantation with a reduced risk of immune-mediated rejection [8].

Tissue engineering paradigms increasingly focus on UC-MSCs obtained from either umbilical cord blood or Wharton’s jelly [8], the latter providing both a structural matrix and a supportive microenvironment for stem cell proliferation [9]. Encouraging preclinical and clinical outcomes—particularly in hematologic and neurological applications [10]—have stimulated further investigation into the regenerative potential of Wharton’s jelly-derived MSCs. These cells demonstrate robust chondrogenic capacity, are readily harvestable and storable, and critically, have not exhibited oncogenic transformation, underscoring their translational potential for future regenerative therapies targeting articular cartilage defects. The distinguishing contribution of this review is its combination of a clinically grounded account of WJ-MSC-based cartilage repair, delivered through a dry-arthroscopic surgical technique with reported patient outcomes, together with an explicit discussion of how AI-assisted bioprinting could next standardize and scale this specific therapeutic approach; prior reviews have generally addressed WJ-MSC biology or bioprinting technology in isolation rather than as a single translational pathway [11,12].

## 2. A Brief History of Mesenchymal Stem Cells in Orthopedics

The use of mesenchymal stem cells (MSCs) for treating early-stage knee osteoarthritis (OA) is a rapidly advancing field within orthopedics. Various techniques are being explored to promote the repair of articular cartilage: including intra-articular injections [11] of biological agents such as platelet-rich plasma (PRP) and concentrates of autologous stem cells derived from bone marrow or adipose tissue [12].

Research on mesenchymal stem cells began in 1966, when Fridenshtein and his team cultured bone-forming cells from the bone marrow and spleen cells of guinea pigs. This marked the first exploration into MSCs [1,2]. The concept of biologically restoring the articular surface of the knee joint was later introduced in 1994 by Caplan and Goldberg [1]. A significant breakthrough came in 2011, when Alberto Gobbi conducted a pioneering clinical study in which he treated patients with early-stage OA by reconstructing degenerative changes in knee cartilage using a hyaluronic acid matrix impregnated with autologous bone marrow concentrate [13].

Initially, these procedures were performed through open surgery, but over time, they evolved to be performed using minimally invasive dry arthroscopy techniques. Further advancements were made, as researchers explored the transplantation of stem cells sourced not only from autologous adipose tissue but also from umbilical cord blood and eventually from the umbilical cord itself [3].

## 3. Understanding Mesenchymal Stem Cells (MSCs)

MSCs have emerged as a promising tool for tissue regeneration due to their ability to self-renew, differentiate into various cell types, modulate immune responses, and enhance tissue repair. MSCs can be isolated from a variety of tissues, including bone marrow, skeletal muscle, synovial membranes, periodontal ligaments, Wharton’s jelly, umbilical cord, umbilical cord blood, amniotic fluid, placenta, and adipose tissue. Recent studies suggest that the therapeutic effects of MSCs are largely mediated by their secreted factors rather than their direct differentiation into specific cell types. These paracrine factors, which include chemokines, cytokines, and growth factors, influence nearby cells, regulate immune responses, and promote tissue regeneration [14]. Through these mechanisms, MSCs can accelerate the healing of various tissues, such as skin, bone, liver, and kidneys, by promoting extracellular matrix remodeling, reducing apoptosis, and enhancing angiogenesis [15,16].

The most common sources for harvesting MSCs are bone marrow aspirate (BMA) and adipose-derived stem cells (ADSCs). The yield of stem cells obtained via liposuction from adipose tissue is significantly higher (1–7%) compared to bone marrow aspiration (0.001–0.02%) [13]. Additionally, harvesting MSCs from adipose tissue is less invasive, with fewer associated side effects compared to BMA. However, bone marrow-derived MSCs exhibit greater morphological and genetic stability, although their cartilage-osteogenic potential is comparable to that of adipose-derived stem cells [17]. Compared with bone marrow- and adipose-derived MSCs, WJ-MSCs additionally offer umbilical cord blood-derived MSCs (UCB-MSCs) as a further allogeneic alternative; however, UCB-MSCs are typically obtained in lower cell yields and show more variable chondrogenic differentiation capacity across donors, whereas WJ-MSCs combine high proliferative capacity, low immunogenicity, and a chondrogenic potential comparable to bone marrow-derived MSCs under three-dimensional culture conditions, without the donor-age-related decline observed in BM-MSCs [18]. Mechanistically, the therapeutic action of WJ-MSCs in cartilage repair is attributed to a combination of paracrine signaling (secretion of trophic growth factors and cytokines that support chondrocyte survival), immunomodulation (suppression of pro-inflammatory T-cell and cytokine responses), direct chondrogenic differentiation, extracellular matrix remodeling, and downregulation of catabolic inflammatory mediators within the joint microenvironment [18]. These mechanisms are not without controversy: in vitro chondrogenic differentiation of MSCs, including WJ-MSCs, frequently proceeds toward a hypertrophic chondrocyte phenotype marked by type X collagen and matrix metalloproteinase-13 expression, raising concern that MSC-derived cartilage may be prone to calcification and endochondral ossification rather than forming stable, durable hyaline cartilage [19]. Reported outcomes are also inconsistent across studies and donors, and inconsistent chondrogenic differentiation, donor-dependent variability in potency, and uncertain long-term durability of the repair tissue remain open, unresolved concerns for the field rather than settled advantages of WJ-MSC therapy.

## 4. Applications of Human Allogeneic Mesenchymal Stem Cells in Orthopedic Disorders

The following Section 4.1, Section 4.2, Section 4.3, Section 4.4, Section 4.5 and Section 4.6 summarize MSC applications across the broader orthopedic and connective-tissue field for contextual completeness; they are presented briefly, as the central focus of this review remains cartilage regeneration, osteochondral repair, and knee osteoarthritis, which are addressed in depth in Section 5, Section 6, Section 7, Section 8 and Section 9.

### 4.1. Management of Bone Fractures

MSCs are typically isolated from bone marrow and can be administered directly to the fracture site, intravenously, or delivered through scaffolds combined with cytokines. Ongoing research is exploring the use of genetically modified MSCs and three-dimensional scaffolds that enable the sustained release of growth factors, potentially enhancing MSC survival and prolonging their regenerative capabilities [18].

Animal studies have demonstrated the efficacy and safety of MSCs from various tissue sources in promoting bone fracture repair. This occurs either through direct differentiation into osteoblasts or by inhibiting inflammatory mediators [19]. A significant milestone was achieved by Marcacci et al., who were the first to demonstrate the long-term success of bone regeneration using tissue engineering. They treated four patients with large bone defects by expanding bone marrow-derived stromal cells in culture and seeding them onto porous hydroxyapatite ceramic scaffolds, which were customized to match the size and shape of the bone defect [20]. Further advances were made by Mazzoni et al., who showed that human mesenchymal stem cells (hMSCs) combined with innovative biomaterials yielded promising results in treating bone injuries. The regenerative potential of hMSCs, coupled with scaffolds that mimic natural bone structure, supports effective healing and bone regeneration [21].

### 4.2. Degeneration of Intervertebral Discs

Intervertebral discs (IVDs) are gel-like fibrocartilaginous structures located between the vertebrae of the spine, functioning as shock absorbers. These discs consist of three main components: the nucleus pulposus, the annulus fibrosus, and the cartilage endplates. Research in both animal models and clinical studies has revealed that each of these components contains cells with stem cell-like characteristics, including specific surface markers, unique morphology, proliferative capacity, and the potential for multilineage differentiation. This discovery suggests that IVDs house stem cells, which could be harnessed for regenerative medicine and tissue engineering applications.

Intervertebral disc degeneration is a chronic condition marked by the loss of proteoglycans and water content in the nucleus pulposus, damage to the annulus fibrosus, and the development of osteophytes. A critical factor in this degeneration is the insufficient production of extracellular matrix (ECM) by disc cells, leading to structural deterioration [22].

Sobajima et al. demonstrated that MSCs migrate to injured disc tissue, where they engraft and promote regeneration by enhancing ECM production. In vitro studies showed that co-culturing MSCs with nucleus pulposus cells (NPCs) significantly increased ECM production. In vivo experiments confirmed the survival of MSCs in rabbit disc tissue for up to 24 weeks, further supporting their role in disc regeneration [23]. In a clinical context, Yoshikawa et al. explored the use of autologous bone marrow-derived MSCs in two patients suffering from low back pain, leg pain, and numbness. The patients reported significant improvements in pain and disability within three months, along with increased disc hydration observed within one year of MSC treatment [19]. These findings highlight the potential of MSC-based therapies for treating intervertebral disc degeneration.

### 4.3. Treatment of Osteoporosis

Osteoporosis is a chronic skeletal disorder characterized by decreased bone mineral density and deterioration of bone microstructure, resulting from an imbalance between bone formation by osteoblasts and bone resorption by osteoclasts. In recent years, stem cell therapies have emerged as a promising approach for promoting bone regeneration in patients with osteoporosis [19].

Multipotent MSCs, found in the bone marrow, have the ability to differentiate into osteoblasts and contribute to bone formation. However, as individuals age, the number of MSCs in the bone marrow declines, and the mechanisms that support bone formation weaken. This reduction in MSCs and their activity is thought to be a key factor contributing to the decrease in bone formation and the increased bone fragility seen in osteoporosis. Therefore, therapies that focus on enhancing the number and/or activity of osteoblasts may provide a more effective way to promote bone formation and regeneration. Stimulating osteogenesis through the activation of MSCs presents a logical therapeutic strategy for preventing osteoporosis [24].

Studies on animal models have shown that both allogeneic and autologous bone marrow-derived mesenchymal stem cell (BMMSC) transplants can be effective in treating osteoporosis. For example, in a mouse model of glucocorticoid-induced osteoporosis, treatment with allogeneic BMMSCs promoted bone formation and increased osteoblast activity. Additionally, in a clinical study led by Lozano-Rivas, patients with recent osteoporotic fractures experienced pain relief following intravenous administration of autologous fucosylated BM-MSCs. Furthermore, stem cells derived from perinatal tissues, including human umbilical cord (hUC), umbilical cord blood (hUCB), amnion, and chorion, have garnered significant interest for their potential to prevent bone loss and aid in the treatment of osteoporosis [19].

These findings highlight the potential of MSC-based therapies for addressing the underlying mechanisms of osteoporosis, offering a novel approach to bone regeneration and fracture healing.

### 4.4. Interventions for Rheumatoid Arthritis

Rheumatoid arthritis (RA) is a chronic autoimmune disorder that affects the entire body, characterized by excessive growth of synovial tissue, which leads to cartilage destruction and systemic complications. The disease is marked by inflammation and hyperplasia of the synovial membrane, the presence of autoantibodies such as rheumatoid factor (RF) and anticitrullinated protein antibodies (ACPA), as well as deformities in both cartilage and bone [10].

In recent years, MSC-based therapies have emerged as a promising approach for treating inflammatory cartilage damage in conditions like RA. Research has shown that MSC treatment reduces the number of harmful T cells in models of collagen-induced arthritis (CIA). Other studies have demonstrated that MSCs contribute to the elimination of activated T cells through the FasL/Fas signaling pathway in OA. Additionally, MSCs help regulate the immune system in RA by inhibiting the production of pro-inflammatory cytokines, such as interferon-gamma (IFN-γ) and tumor necrosis factor-alpha (TNF-α), as well as matrix-degrading enzymes like collagenase and gelatinase.

Animal studies investigating the use of BM-MSCs in models of induced RA have shown significant reductions in inflammation, joint swelling, and cartilage damage compared to untreated animals. In a clinical trial, Park and his team were the first to explore the use of human umbilical cord blood-derived mesenchymal stem cells (hUCB-MSCs) in patients with RA. The results showed no severe side effects or abnormalities in blood tests, both during and after treatment [19].

These findings highlight the potential of MSC-based therapies not only to reduce inflammation and immune dysfunction but also to protect cartilage and bone in patients with RA, offering a novel therapeutic strategy for managing this debilitating autoimmune disease.

### 4.5. Addressing Osteogenesis Imperfecta

Osteogenesis imperfecta (OI), commonly referred to as “brittle bone disease,” is a rare inherited skeletal disorder characterized by bone fragility, skeletal deformities, and short stature. It affects approximately 1 in 15,000 to 20,000 live births. Beyond its primary impact on bones, OI also influences other connective tissues, causing additional symptoms such as dentinogenesis imperfecta, hearing loss, joint hypermobility, blue sclera, basilar invagination, and abnormalities in the cardiovascular and respiratory systems [25].

Recent research has shown promising results using MSCs for treating OI through intrauterine or multi-site implantation in both animal models and humans. Studies have demonstrated that both human fetal (HF) and adult MSCs can successfully differentiate into functional osteoblasts in OI models. Le Blanc et al. conducted a notable study in which allogeneic human fetal mesenchymal stem cells (hf-MSCs) were transplanted intrauterine into a female fetus with OI at 32 weeks of gestation. The results indicated that the transplanted hfMSCs were able to differentiate into bone within the fetus, suggesting potential for in utero intervention. Additionally, the transplantation of allogeneic BMMSCs in children with OI showed successful cell retention in various sites, including bones, skin, and bone marrow stroma, with a significant increase in growth rate observed within six months of treatment [19].

The use of MSCs from bone marrow in OI patients has been shown to increase bone mineral density, which, in turn, reduces the frequency of fractures [22]. These findings highlight the potential of MSC-based therapies as a promising treatment option for improving bone strength and overall outcomes in patients with OI.

### 4.6. Tendon Repair and Regeneration

Tendons play a critical role in facilitating movement but have limited self-healing capacity due to poor vascularization. MSCs have been shown to support tendon regeneration by promoting cell proliferation and angiogenesis. Research indicates that MSC transplantation accelerates the healing of injured tendons, particularly in the patellar tendon [22].

In the field of regenerative medicine, there has been increasing interest in the use of stem cell-derived secretory products, such as secretomes and exosomes, for tendon and bone regeneration. The secretome, which includes conditioned medium (CM) rich in bioactive molecules such as cytokines and growth factors (e.g., VEGF, PDGF, TGF), plays a vital role in stimulating tissue repair by promoting cell proliferation, migration, and differentiation. Several recent studies have demonstrated the positive effects of secretomes or CM on tendon and bone regeneration, particularly in animal models involving cruciate ligament transplantation or rotator cuff repair in rats [4].

These findings underscore the potential of MSC-derived products not only in direct cell therapy but also in enhancing tissue repair through the bioactive molecules they secrete, making them promising tools in the treatment of tendon injuries and bone regeneration.

## 5. Utilizing MSCs for Osteoarthritis Treatment and Cartilage Regeneration

OA is one of the most prevalent musculoskeletal disorders, significantly contributing to disability among older adults. The condition presents a treatment challenge due to the limited capacity of joint cartilage to regenerate and the absence of specific diagnostic biomarkers [26].

Cartilage is a specialized form of connective tissue characterized by a unique ECM composition. It is avascular, lacking blood vessels, lymphatics, and nerves, and relies on diffusion from surrounding tissues for nourishment, resulting in limited self-repair capabilities. Cartilage is classified into three types: hyaline cartilage, fibrocartilage, and elastic cartilage [27]. Among these, hyaline cartilage is the most common, covering the articular surfaces of joints. Its primary function is to provide a smooth, lubricated surface that facilitates bone movement within the joint while minimizing friction and allowing load transmission. Chondrocytes, spherical cells embedded in the cartilage matrix, maintain the structure of cartilage and remodel its biochemical composition in response to changes in the mechanical and chemical environment, thereby regulating cartilage homeostasis. However, as chondrocytes age, their responsiveness to growth factors diminishes, leading to accelerated cartilage degradation, damage to the cartilage matrix, and the onset of related diseases. Additional contributors to cartilage damage include injuries, specific diseases, and continuous mechanical stress. Consequently, the limited capacity for cartilage self-repair renders the treatment of cartilage damage a significant clinical challenge [19].

Numerous studies have confirmed the efficacy of MSC transplants in treating and repairing damaged cartilage. MSCs contribute to cartilage regeneration by secreting bioactive factors that prevent degradation and by differentiating into chondrocytes [2,27].

Murphy et al. demonstrated that stem cells can regenerate joint cartilage in mice, irrespective of the animals’ age. Their research involved labeling cells in mice of varying ages, from newborns to adults, revealing a significant decrease in the number of stem cells in cartilage with age, from 95.9% in newborns to 57.6% in adult mice. Subsequently, the authors utilized microfracture (MF) to examine how resident cartilage stem cells respond to injury. They observed the emergence of active, dividing cells in the damaged areas, suggesting that the increase in stem cells at the injury site resulted from local expansion rather than recruitment from other body regions, as confirmed by experiments using parabiont mice (GFP/non-GFP) [28].

Akgun et al. compared the effectiveness of two techniques for treating cartilage defects in the knee: autologous matrix-induced stem cell implantation (m-AMI) and autologous matrix-induced chondrocyte implantation (m-ACI). The study included 14 patients with defects exceeding 2 cm^2^, who were assessed preoperatively and at 24 months postoperatively. Results indicated that m-AMI yielded superior functional improvement, enhanced patient experience, and higher quality of life compared to m-ACI, with no graft failures detected via MRI at the 24-month mark [2].

Freitag et al. investigated the efficacy of autologous adipose tissue stem cell (ADMSC) therapy for knee osteoarthritis in a study involving 30 subjects divided into three groups: one receiving a single ADMSC injection, another receiving two injections, and a control group undergoing conservative treatment. After 12 months, both ADMSC treatment groups showed significant improvements in pain and function, as measured by the KOOS and WOMAC scores, without severe side effects. In the control group, 67% of participants experienced progressive cartilage loss, compared to 30% in the one-injection group and 89% in the two-injection group, who showed improvement or no progression in cartilage loss [29].

Matas et al. randomized 29 patients with symptomatic knee OA into three groups: the control group received hyaluronic acid (HA) at baseline and again after six months, while the other two groups received either a single injection of umbilical cord mesenchymal stem cell (UC-MSC) (MSC-1) or repeated injections (MSC-2) after six months. After 12 months, patients receiving stem cell treatment, particularly those in the MSC-2 group, exhibited significant improvements in pain and function, assessed by WOMAC and VAS scales. The MSC-2 group reported lower pain levels and better functional scores compared to the HA group, with no serious adverse events, although differences in MRI results were not statistically significant [30].

Wakitani et al. employed autologous bone marrow MSCs to repair osteochondral defects in both animal and human models, yielding promising outcomes. The MSC transplantation group achieved superior arthroscopic and histological scores compared to controls, highlighting the potential of autologous MSCs in treating early OA. Additional studies have reinforced the benefits of MSC transplantation for cartilage repair, emphasizing the importance of utilizing autologous cell sources for optimal outcomes [6].

A novel approach to articular cartilage repair was introduced by Gobbi, who utilized bone marrow aspirate concentrate (BMAC) containing MSCs combined with a hyaluronic acid-based material. This single-step procedure eliminates the need for cell culture, significantly reducing treatment costs while demonstrating positive long-term outcomes in patients over 45 years of age. Concentrated BMAC may also contain hematopoietic progenitor cells, further enhancing its regenerative potential [31,32,33].

Moreover, synovial tissue has emerged as a promising cell source, with Jorgenson et al. developing a suspension bioreactor that employs microcarrier technology to enhance the cultivation of MSCs derived from synovial fluid [30]. Shimomura et al. reported success in utilizing scaffold-free tissue-engineered constructs derived from autologous synovia-derived MSCs for the repair of knee chondral lesions, demonstrating safety and efficacy in treating articular cartilage defects [3,11].

These studies collectively underscore the transformative potential of MSC-based therapies in addressing osteoarthritis and cartilage repair, presenting innovative strategies to improve patient outcomes and quality of life.

## 6. Wharton’s Jelly-Derived Mesenchymal Stem Cells (WJ-MSCs)

Wharton’s Jelly, first identified by Thomas Wharton in 1656, is a gelatinous substance found within the umbilical cord, providing structural support and protection for the umbilical blood vessels. This unique tissue is rich in mucopolysaccharides like hyaluronic acid and chondroitin sulfate and contains various cell types, including fibroblasts, myofibroblasts, and collagen fibers. Mesenchymal stem cells derived from Wharton’s Jelly (WJ-MSCs) are particularly notable for their high proliferation rate, multipotency, and immunomodulatory properties, which make them excellent candidates for applications in regenerative medicine [34,35].

**Figure 1:**
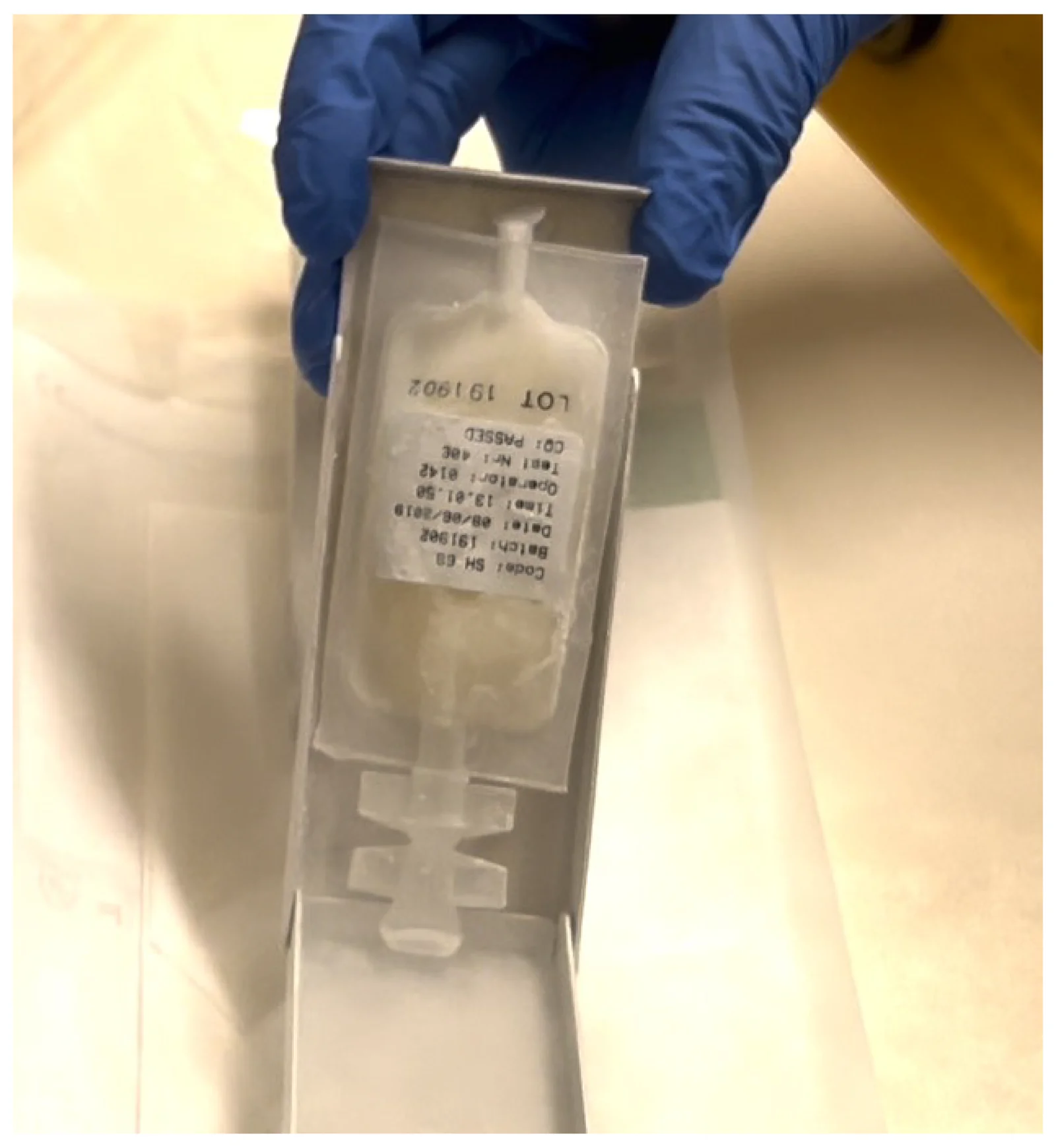
Isolated and suspended in a mixture containing human albumin and 10% dimethyl sulfoxide WJ-MSCs in a freezing bag delivered to the OR.

There are two primary types of MSCs derived from the umbilical cord: human mesenchymal stem cell from umbilical cord blood (UCB-MSCs) and WJ-MSCs. UCB-MSCs are obtained from the blood of the umbilical cord and require immediate collection after birth, often involving complex separation processes that result in lower concentrations of MSCs [36]. In contrast, WJ-MSCs are isolated from the gelatinous Wharton’s Jelly using less invasive techniques, leading to higher concentrations of viable MSCs (Figure 1) [8,37].

Both types of MSCs express standard mesenchymal stem cell markers, including CD73, CD90, and CD105, while lacking hematopoietic markers such as CD34 and CD45. However, WJ-MSCs generally demonstrate superior proliferation rates and broader differentiation potential into osteogenic, chondrogenic, adipogenic, and myogenic lineages, which can be attributed to their more primitive and less differentiated nature [14].

Compared to mesenchymal stem cells derived from adipose tissue (AD-MSCs) and BMMSCs, WJ-MSCs and UCB-MSCs offer several advantages. WJ-MSCs grow faster, can be expanded more efficiently in culture, are often purer, and can be collected without invasive procedures, making them more readily available for therapeutic use. Additionally, they can be used repeatedly in treatments, increasing their utility in clinical applications [19].

Both WJ-MSCs and UCB-MSCs exhibit significant immunomodulatory effects, which are crucial for therapies involving allogeneic transplants. Research suggests that WJ-MSCs may possess stronger immunosuppressive capabilities than UCB-MSCs, attributed to their higher expression of immunomodulatory factors. This characteristic makes WJ-MSCs potentially more effective in treating inflammatory and autoimmune diseases [38].

In a study conducted by Amable et al., WJ-MSCs demonstrated enhanced proliferation potential, which was linked to their increased production of pro-inflammatory cytokines such as IL-6, as well as higher expression levels of growth factors including PDGF, HGF, and TGF-β. Furthermore, WJ-MSCs were found to produce greater amounts of pro-angiogenic proteins, extracellular matrix components, and matrix metalloproteinases (MMPs), further highlighting their advantageous role in tissue regeneration and repair [39].

In summary, Wharton’s Jelly-derived MSCs represent a promising resource for regenerative medicine due to their favorable biological properties, including rapid proliferation, broad differentiation capabilities, and significant immunomodulatory effects. These characteristics position WJ-MSCs as a vital tool in advancing therapies for a range of diseases, particularly those involving inflammation and tissue damage.

## 7. Investigating Articular Cartilage Regeneration with Wharton’s Jelly Stem Cells

### 7.1. In Vitro and Animal Studies

Chen et al. isolated MSCs from human umbilical cord Wharton’s Jelly to explore their potential in promoting cartilage healing. The researchers cultured the UC-MSCs within a collagen hydrogel, stimulating them to differentiate toward chondrogenesis. This differentiation was assessed using various techniques, including PCR, histochemistry, and immunohistochemistry, while also monitoring cell viability and apoptosis. The study found that UC-MSCs effectively differentiated into chondrocytes, evidenced by an increased expression of key cartilage markers. Notably, after three weeks, a majority of the UC-MSCs maintained their viability, indicating that collagen hydrogel can support their chondrogenic differentiation. This suggests a promising role for UC-MSCs in cartilage engineering applications [27].

Kusuma et al. investigated the effects of conditioned medium (CM) derived from WJ-MSCs that were treated with insulin-like growth factor 1 (IGF1) on OA in the knee. Their findings revealed that this conditioned medium significantly enhanced the proliferation and viability of chondrocytes, while also promoting the production of critical cartilage proteins, including aggrecan and type II collagen. Furthermore, the CM with IGF1 demonstrated a protective effect against cartilage degradation by activating specific signaling pathways essential for maintaining cartilage homeostasis [5].

In another study, Muthuchamy and colleagues examined the use of decellularized human umbilical cord Wharton’s Jelly (hUC-WJ) as a biomaterial for cartilage tissue engineering. Their results indicated that the hUC-WJ scaffold effectively supported the attachment, viability, and transfer of autologous chondrocytes without altering their phenotype. This finding highlights the potential of hUC-WJ as a viable material for cartilage regeneration, particularly due to its accessibility and its biochemical similarities to the extracellular matrix of cartilage [40].

Aleksander-Konert et al. conducted research using hydrogel scaffolds to evaluate the growth capacity of WJ-MSCs and chondrocytes, as well as their metabolic activity. They assessed the chondrogenic differentiation of WJ-MSCs through staining methods, including Alcian blue and Safranin O, alongside PCR analysis to evaluate the expression of collagen types I, II, III, and aggregating proteoglycan genes. The study found that the average survival rate of WJMSCs and chondrocytes on the hydrogel scaffolds was approximately 67%. Staining results indicated a significant production of an extracellular matrix rich in proteoglycan, which is a primary component of hyaline cartilage. Additionally, there was an increased expression of collagen type II and aggrecan, further supporting the notion that WJ-MSCs have the capability to differentiate into chondrocytes [41].

These studies collectively underscore the potential of Wharton’s Jelly-derived MSCs and their conditioned media in cartilage regeneration and highlight the promising applications of this tissue in regenerative medicine, particularly for osteoarthritis and other cartilage-related conditions.

### 7.2. Clinical Trials and Outcomes

The regenerative potential of hUC-MSCs in cartilage repair has been the focus of extensive research, particularly regarding their efficacy in enhancing function and alleviating pain in patients suffering from degenerative changes in the knee joint. One of the distinguishing features of hUC-MSCs is their relatively young cellular age, which contributes to their greater proliferative capacity and pluripotency compared to adult stem cells. This characteristic positions them as promising candidates for regenerative therapies [42,43].

The ECM components found in Wharton’s jelly are remarkably similar to those present in cartilage. Human Wharton’s jelly-derived MSCs (hWJ-MSCs) produce key cartilage-related proteins, such as aggrecan, type II collagen, and SOX-9, akin to the cells found in cartilage tissue. Moreover, hWJ-MSCs secrete growth factors, chemokines, and cytokines in amounts comparable to those in cartilage, indicating their potential utility in cell therapy for OA. Animal studies have demonstrated that human MSCs derived from hUCB-MSCs have a lower likelihood of eliciting an immune response and a superior capacity to differentiate into cartilage tissue, promoting cartilage repair over extended periods without leading to unwanted bone formation [19].

In a clinical trial conducted by Yusof et al., the safety and efficacy of WJ-MSC injections were evaluated in patients with knee osteoarthritis. This trial involved 11 subjects aged between 42 and 71 years, with eight patients receiving WJ-MSCs in one knee and three in both knees. Over a monitoring period of six months, patients were injected with 15 × 10^6^ WJ-MSCs in a 2 mL hyaluronic acid saline solution. The careful preparation and characterization of WJ-MSCs ensured their viability when cryopreserved for later use. The injection procedure was found to be safe, with transient knee pain and swelling being the most common adverse effects reported. Remarkably, significant improvements were observed in pain levels, knee function, and overall quality of life after 24 weeks, as indicated by improvements in KOOS and IKDC scores. Additionally, there was a notable reduction in the requirement for pain medication, and minor radiological changes were documented, including an increase in the joint space width by 2% in the medial compartment and 8% in the lateral compartment [44].

Another study by Gunay et al. examined the effects of WJ-MSC injections in 10 patients with knee OA who had not responded to conservative treatment over six months. The findings revealed reduced pain levels on the VAS scale, alongside improved function and quality of life as measured by the WOMAC and SF-36 scales. MRI assessments using T2* cartilage mapping indicated a decrease in cartilage quality in nine joint regions and an increase in five regions. Notably, cartilage thickness showed significant improvement in five areas, highlighting the potential of WJ-MSCs in enhancing cartilage repair [45].

Samara et al. aimed to evaluate the impact of WJ-MSC injections in treating advanced knee OA. This study included 16 patients who received two doses of WJ-MSCs at one-month intervals. After 48 months of follow-up, significant improvements were noted in KOOS and VAS scores, alongside MRI scans that demonstrated marked recovery in cartilage integrity, osteophytes, and bone marrow lesions at the 12-month mark. The results indicated a shift in the severity of cartilage damage over time, with more patients transitioning from moderate or severe damage to mild conditions, demonstrating the potential long-term benefits of WJ-MSC therapy [45].

In a retrospective analysis, Pałka et al. compared two techniques for addressing cartilage defects in the knee: one utilizing matrix impregnated with bone marrow aspirate concentrate-derived mesenchymal stem cells (BMAC-MSCs) and the other using hUCB-MSCs. Thirty-nine patients were assessed over 12 months using various scales, including VAS, KOOS, and Lysholm, as well as through radiological evaluations. Both treatment modalities resulted in significant improvements in quality of life, with no notable differences between the two groups, despite the hUCB-MSC group comprising older patients with larger defects. Both techniques were deemed safe and effective, indicating a promising avenue for future clinical trials [43].

**Figure 2:**
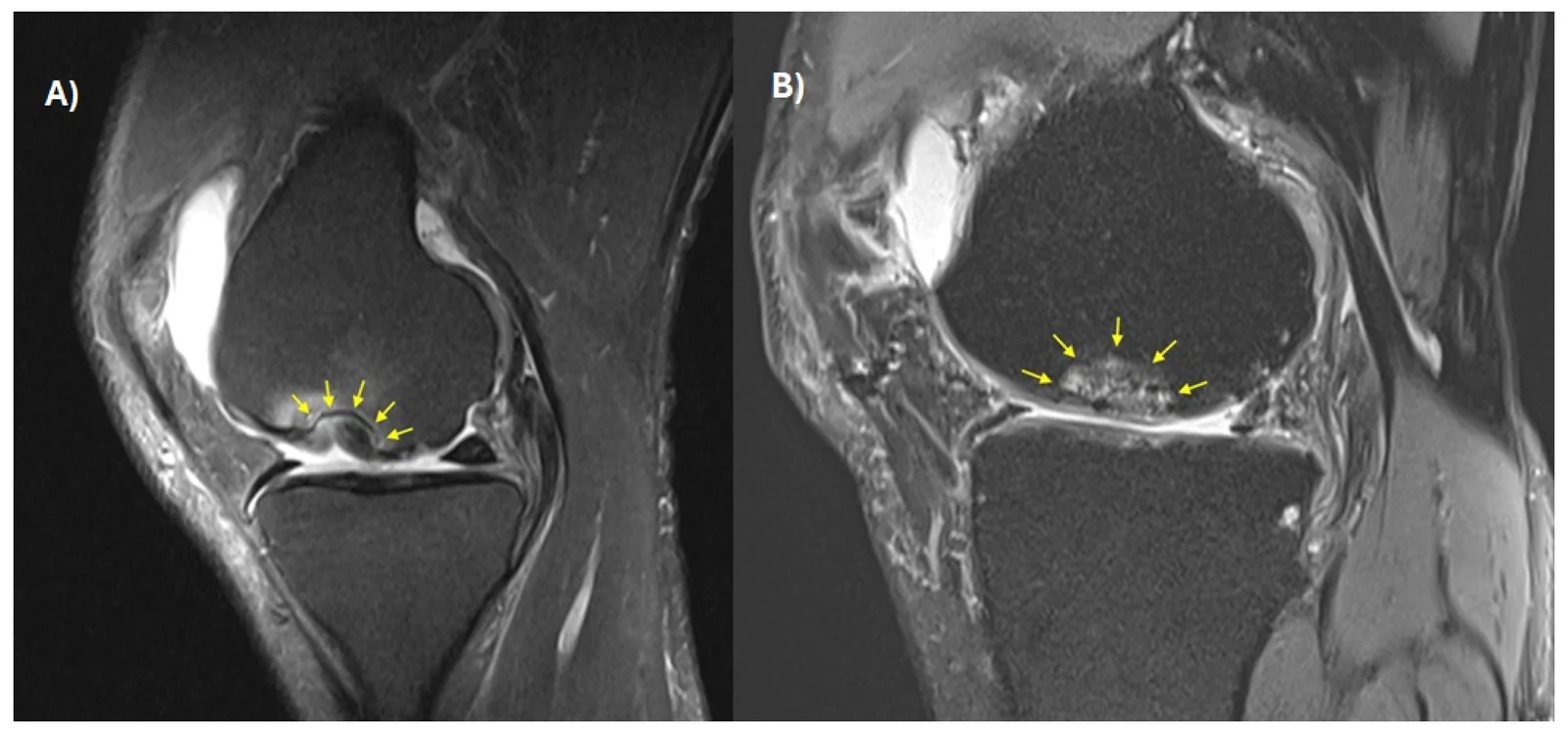
(**A**) The spot of osteonecrosis of the medial femoral condyle (MFC) visualized in MRI using PD Fat Sat sequence. (**B**) The spot of regenerated osteochondral tissue at MFC visualized in MRI using PD Fat Sat sequence 7 years after implantation autologous morselized bone chips and a collagen scaffold soaked with WJ-MSCs in dry arthroscopy technique.

Collectively, these studies underline the promising role of human umbilical cord MSCs, particularly those derived from Wharton’s jelly, in cartilage repair and the potential of these cells in treating osteoarthritis (Figure 2).

## 8. Implantation of WJ-MSCs in Scaffolds Using Dry Arthroscopy for Regenerating Large Joint Surface Lesions

Allogeneic human umbilical cord mesenchymal stem cells (hUC-MSCs) derived from WJ-MSCs exhibit remarkable viability and growth potential, making them an attractive option for regenerative therapies. The ability to store these cells through cryopreservation ensures consistent quality for clinical applications, which is essential for their effective use in treatment.

**Figure 3:**
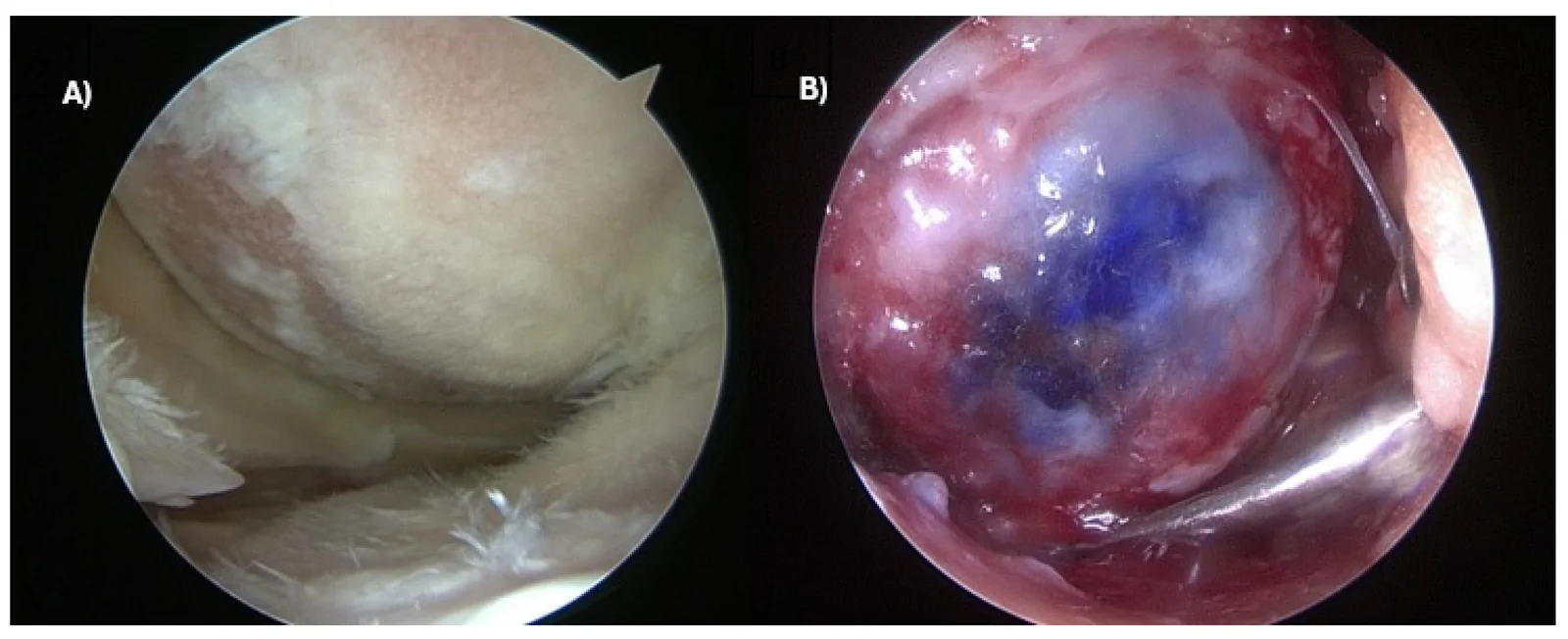
(**A**) Spot of osteonecrosis of the medial femoral condyle (MFC) visualized in the arthroscopic camera. (**B**) The final position of implanted scaffolds/matrices embedded with WJ-MSCs covered by fibrin glue.

A significant advancement in the delivery of hUC-MSCs to articular cartilage defects is the utilization of a “single-step” dry arthroscopy technique (Figure 3A). This minimally invasive approach allows for the direct administration of stem cells to the site of injury while minimizing exposure to saline, which could otherwise dilute the cells and potentially reduce their effectiveness. By employing this technique, clinicians can enhance the likelihood of successful cell engraftment and function within the cartilage [46]. This technique is primarily indicated for focal, contained chondral and osteochondral lesions of the femoral condyles and trochlea, and is generally reserved for patients in whom bone marrow stimulation techniques have failed or are considered unlikely to succeed; scaffold selection is guided by lesion depth and containment, while postoperative protocols typically involve a period of protected weight-bearing followed by progressive rehabilitation to support scaffold integration and cell retention under physiological joint loading.

In addition to direct injection, another effective strategy in treating cartilage damage involves embedding hUC-MSCs within biomaterials designed to mimic the ECM (Figure 3B). These biomaterials play a crucial role in promoting cell growth and differentiation, as they provide a supportive environment that facilitates cell adhesion and retention at the injury site. By mimicking the natural ECM, these materials help create an optimal niche for the stem cells, enhancing their regenerative capabilities and improving overall treatment outcomes for cartilage repair [47].

Table 1 summarizes recent clinical trials investigating the therapeutic potential of WJ-MSCs for articular cartilage regeneration and the treatment of knee osteoarthritis. Over the past decade, several early-phase clinical studies have explored the safety, feasibility, and preliminary efficacy of intra-articular WJ-MSC administration. Most trials have focused on patients with symptomatic knee osteoarthritis, evaluating clinical outcomes using standardized functional and pain-related scales such as WOMAC, KOOS, IKDC, and VAS, often complemented by imaging-based cartilage assessment using MRI. The available evidence consistently indicates a favorable safety profile, with phase I studies reporting no serious treatment-related adverse events and good tolerability following intra-articular cell delivery. Moreover, several prospective studies have demonstrated clinically meaningful improvements in pain, joint function, and patient-reported outcomes during follow-up periods of up to 12 months. However, despite these encouraging findings, many trials remain ongoing or have not yet reported final results, and most studies involve relatively small patient cohorts typical of early-phase translational research. Consequently, larger randomized controlled trials with longer follow-up are required to confirm the regenerative potential of WJ-MSC–based therapies and to establish standardized protocols for cell preparation, dosing, and clinical application in cartilage repair. It is important to distinguish between different tiers of evidence reported across these studies: most trials to date substantiate safety and short-to-medium-term symptomatic improvement (pain and functional scores), a smaller subset report favorable MRI-based structural changes (e.g., defect filling, cartilage surface quality), while direct histological or biopsy-confirmed evidence of true hyaline cartilage regeneration in humans remains limited. Furthermore, the current evidence base is constrained by small sample sizes (typically 10–16 patients per study), heterogeneous and non-standardized outcome measures (VAS, WOMAC, KOOS, IKDC, SF-36 used inconsistently across trials), short follow-up periods in most studies, and the general absence of placebo or active-comparator control arms; these limitations should be considered when interpreting the reported efficacy signals.

Integrating these technological innovations with the unique biological properties of Wharton’s jelly-derived MSCs highlights the convergence of two complementary paradigms: biology-driven regeneration and technology-driven standardization. While AI-assisted bioprinting provides predictive design, quality control, and reproducibility, WJ-MSCs contribute the essential cellular and immunomodulatory components needed for durable cartilage repair [34]. The synergy between advanced computational platforms and allogeneic stem cell biology thus sets the stage for translational strategies that are both scientifically robust and clinically applicable, reducing variability while moving closer to scalable, patient-tailored therapies.

## 9. Multimodal AI-Assisted Bioprinting: From Material Selection to Automated Fabrication

Having reviewed the clinical evidence for WJ-MSC-based cartilage repair above, the remainder of this review shifts focus to prospective manufacturing technology: the rapid evolution of 3D bioprinting is beginning to transform how musculoskeletal tissues are reconstructed, particularly in orthopedics where composite grafts must simultaneously replicate cartilage, bone, and tendon/ligament biomechanics. Despite progress, current methods remain constrained by empirical trial-and-error in bioink formulation, limited real-time monitoring during fabrication, and inconsistent reproducibility across laboratories. Artificial intelligence (AI) and multimodal imaging technologies offer a paradigm shift: enabling predictive bioink design, closed-loop quality control, and ultimately autonomous bioprinting workflows that can be standardized for clinical translation. Recent 2025–2026 literature on cartilage- and osteochondral-specific bioprinting confirms this trajectory: reviews of extrusion, inkjet, stereolithography, and digital-light-processing approaches for cartilage regeneration converge on AI-guided bioink optimization and gradient scaffold design as central near-term priorities, reinforcing that the AI-assisted strategies discussed below are considered specifically in relation to cartilage and osteochondral repair rather than as a generic bioprinting overview [48].

### 9.1. AI-Driven Bioink Selection and Predictive Design

Bioink design for orthopedic use must balance mechanical fidelity with biological compatibility. Hydrogel-based inks, for example, often trade stiffness for cell viability: higher crosslinking enhances shape fidelity but can impair nutrient diffusion and cell proliferation. Recent reviews emphasize that rheological parameters such as viscosity, shear-thinning, and yield stress critically determine extrusion consistency and filament fusion [48,49]. Notably, cell density itself is a rheological modifier: Diamantides et al. demonstrated that high cell seeding in collagen bioinks altered viscoelastic properties and printability, with implications for cartilage grafts where cell-rich environments are essential [42].

Machine learning approaches are emerging as a powerful tool to predict printability from bulk rheological data [50]. Nadernezhad et al. showed that supervised ML models could identify generalized relationships between elastic modulus, yield stress, and extrusion behavior across diverse hydrogel systems [51]. Similarly, Yu et al. applied ML to optimize polymer bioinks, reducing experimental iterations and improving structural fidelity [52]. Building on these findings, AI can be trained to generate “bioprintability maps” that guide formulation choices based on intended application—soft viscoelastic cartilage, mineralized subchondral bone, or fibrocartilaginous tendon-bone interfaces [52]. Among these approaches, random forest algorithms have shown particularly strong and consistent performance: in a benchmark comparison across 210 biomaterial formulations, a random forest model achieved the highest accuracy (88.1%), precision (90.6%), and F1 score (87.0%) among decision-tree, random-forest, and deep-learning classifiers, while the deep-learning model achieved the highest recall (87.3%) [53]. More recent rheology-informed hierarchical neural network models have further improved prediction of printing resolution and positional error in suspended bioprinting relative to classical random forest and support vector regression baselines [54]. Importantly, these frameworks have begun to move beyond predicting structural/geometric fidelity alone: neural-network-based Bayesian optimization models have now been trained to predict post-extrusion cell viability directly from shear-stress-related printing parameters [54], although such viability-prediction models remain validated mainly on generic gelatin/alginate or alginate/hyaluronate systems and have not yet been demonstrated specifically for WJ-MSC-laden bioinks. Concretely for WJ-MSCs, AI-guided approaches could be used to identify bioink formulations and print parameters that preserve WJ-MSC viability under extrusion shear stress, tune scaffold stiffness gradients to match the biomechanical demands of the specific implantation site, and predict construct-level compressive modulus before implantation. This last point matters because cartilage regeneration is fundamentally a biomechanical problem, not only a biological one: native articular cartilage exhibits a much higher tensile than compressive modulus and depends on interstitial fluid pressurization for low-friction load transmission, and tribological performance (coefficient of friction, wear resistance) is a critical but frequently overlooked release criterion for engineered cartilage, alongside the mechanical integration of the construct with surrounding native tissue at the repair margin [52,55]. AI-assisted bioink and structure optimization for WJ-MSC-laden constructs should therefore explicitly target these biomechanical and tribological benchmarks, not structural fidelity alone.

Translational innovation for orthopedics: Such predictive frameworks could integrate mineralization parameters (e.g., calcium-phosphate nanoparticle fraction, ion release kinetics) as input features. This would enable the design of graded osteochondral bioinks, with cartilage-like viscoelasticity in superficial zones and bone-like stiffness in deeper layers, reproducing the mechanical transitions of the native osteochondral unit [48,56].

### 9.2. Multimodal Imaging for In-Situ Monitoring

While AI-driven material selection improves the input quality, real-time monitoring during fabrication is essential to ensure fidelity of the printed construct. Traditional post-print imaging (e.g., micro-CT) is too slow for error correction. Instead, label-free, real-time imaging modalities such as optical coherence tomography (OCT) and photoacoustic imaging (PAI) have gained prominence [31].

OCT provides micron-scale resolution and has been integrated into extrusion systems to detect filament collapse, layer misalignment, and structural defects [56]. This allows in-process corrections, such as adjusting extrusion pressure or path, thereby reducing failed prints. PAI, in contrast, offers greater imaging depth and the ability to visualize hemoglobin-mimicking absorbers or exogenous vascular contrast agents [57]. This is particularly important in large orthopedic grafts, where monitoring the formation of perfusable channels is essential to overcome diffusion limitations.

Translational innovation for orthopedics: We envision a tri-modal feedback system—OCT for geometry validation, PAI for vascular channel monitoring, and ML-inferred elastography (from OCT speckle dynamics) for near-real-time modulus mapping. A key current limitation is real-time latency: OCT offers high spatial resolution but is constrained by limited depth penetration and computationally intensive processing [56], while photoacoustic imaging historically required point-by-point sequential sensor readout that produced scan times on the order of minutes in early clinical scanners, making it too slow for closed-loop correction during continuous extrusion; more recent parallelized, all-optical PAI architectures have reduced acquisition to seconds or sub-second timescales, but dedicated latency benchmarks within an active bioprinting feedback loop have not yet been published [57]. Such multimodal feedback would allow automated adjustment of crosslinking energy, filament overlap, or nozzle path to ensure zone-specific stiffness (e.g., tendon midsubstance vs. enthesis; superficial vs. deep cartilage) [36].

### 9.3. Closed-Loop Autonomy and Self-Correction

The integration of AI with multimodal imaging paves the way for closed-loop autonomous fabrication. In this paradigm, imaging data (perception) are continuously fed into ML algorithms that classify defects, predict structural deviations, and suggest corrective actions (decision-making). These are then implemented instantly by adjusting extrusion pressure, nozzle speed, or crosslinking dose (actuation) [12].

Proof-of-principle studies have demonstrated AI-assisted defect detection and OCT-guided correction during printing [56]. Extending these systems, orthopedic constructs could be fabricated under surgeon-in-the-loop autonomy, where AI manages micro-scale corrections (e.g., layer thickness, filament fusion) while the surgeon defines macro-scale geometry based on patient anatomy. Such hybrid autonomy is particularly attractive for intraoperative bioprinting, where real-time correction of tissue movement, bleeding, or surface irregularities is critical [58].

### 9.4. Digital Twins for Predictive Fabrication

The concept of a bioprinting digital twin—a dynamic, data-driven replica of the bioprinting process—has gained traction in advanced manufacturing. For musculoskeletal constructs, a digital twin would link rheological input data, imaging-derived structure maps, and mechanical models of load-bearing performance. By synchronizing OCT/PAI monitoring with finite element simulations, the digital twin could predict whether a construct will withstand physiological loads (e.g., gait-induced shear in osteochondral plugs) and adjust subsequent layers accordingly [14,37].

### 9.5. Allogeneic Cell-Based Bioprinting (With Wharton’s Jelly MSCs)

The concept of allogeneic cell-based bioprinting, particularly using WJ-MSCs, is increasingly regarded as both biologically feasible and technologically promising. WJ-MSCs offer a unique profile that makes them highly suitable for regenerative applications: they are relatively immunoprivileged, exhibit robust proliferative capacity, and can be readily banked and expanded under GMP conditions. These characteristics support the vision of “off-the-shelf” biofabricated products, contrasting with autologous constructs that require patient-specific harvesting, culture, and expansion. The immunological safety of allogeneic WJ-MSCs has been supported by clinical data, particularly in osteoarthritis, where intra-articular injections of allogeneic umbilical cord or Wharton’s jelly MSCs have demonstrated both safety and meaningful improvement in pain and cartilage quality compared with conventional therapies. Early-phase trials, including randomized controlled studies, provide a clinical rationale for extending these cells into biofabricated tissue constructs intended for more complex osteochondral repair [45].

From a technical perspective, the use of WJ-MSCs in 3D bioprinting raises important considerations for bioink formulation and construct design [46]. Cartilage and osteochondral tissues require gradient structures that reproduce zonal differences in mechanical stiffness and biochemical composition. Recent studies in cartilage bioprinting have highlighted how rheological parameters—such as viscosity, yield stress, and shear-thinning behavior—directly influence print fidelity, filament fusion, and long-term stability of cell-laden constructs. High cell densities typical of MSC-based inks can significantly alter viscoelasticity, necessitating predictive models to anticipate printability. Here, artificial intelligence and machine learning tools have already demonstrated value by correlating rheological indices with print outcomes, allowing for rapid optimization without exhaustive trial-and-error experiments [51,53,54].

Equally critical is the issue of in-process monitoring and quality control. The integration of real-time imaging modalities such as OCT and PAI provides direct feedback on filament architecture, pore geometry, and the formation of vascular-like channels. When combined with machine learning-driven feedback loops, such multimodal systems create a “digital twin” of the bioprinted construct, continuously predicting its structural integrity and mechanical performance during the fabrication process [48]. This convergence of biological and technological systems may ultimately reduce variability and increase clinical reliability.

Nevertheless, clinical translation of allogeneic bioprinted constructs must proceed under the regulatory framework for advanced therapy medicinal products (ATMPs). The 2025 EMA guideline on investigational ATMPs emphasizes the necessity of Quality-by-Design (QbD) approaches, requiring clear definitions of critical quality attributes such as cell viability, mechanical modulus, filament diameter tolerance, and sterility assurance [58]. This implies that bioprinted constructs with allogeneic WJ-MSCs will need to be produced in GMP-compliant facilities, with validated release assays for phenotypic stability, sterility, and immunogenicity. Beyond release testing, commercial translation of bioprinted WJ-MSC constructs will also depend on manufacturing scalability (moving from single-unit, benchtop bioprinting to reproducible multi-unit production), batch-to-batch reproducibility of both the cell source and the printed construct, and the cost-effectiveness of GMP-compliant, imaging-integrated bioprinting relative to existing surgical techniques, each of which represents a distinct regulatory approval hurdle beyond the cell-therapy framework alone. Immunomonitoring during early clinical trials will be essential to confirm that allogeneic constructs retain the expected low immunogenic profile [59]. In parallel, the 2025 EMA guideline explicitly requires Quality-by-Design documentation of critical quality attributes across the manufacturing chain, which for bioprinted allogeneic constructs will need to extend beyond conventional cell-therapy release criteria to construct-level parameters such as post-thaw/post-print viability and shear-exposure history during fabrication [57,58].

The cost of developing such products remains substantial, driven by GMP-compliant cell banking, clinical-grade bioinks, advanced imaging integration, and regulatory documentation. Early development costs are likely to resemble those of other ATMPs, such as CAR-T cells, but economic models suggest that scalable, allogeneic “off-the-shelf” products could become more cost-efficient over time as automation and standardization reduce per-implant expenses [60].

A further, currently unresolved barrier is cryopreservation: unlike simple cell suspensions, which can retain over 85% post-thaw viability at several hours with optimized DMSO-free protocols, 3D bioprinted constructs face compounded challenges from cryoprotectant diffusion, thermal gradients, and ice formation across their structured geometry, and historically achieved viability below 50% with scaffold sizes limited to under 0.15 cm^3^; newer temperature-controlled cryoprinting and glycerol-based cryobioprinting bioinks have improved this to roughly 70–80% viability, but this still lags behind suspension-based benchmarks and remains a practical obstacle to shelf-stable, off-the-shelf allogeneic products [60]. Shear stress imposed by the extrusion nozzle is a second, well-characterized limitation: viability for mesenchymal stromal/stem cells declines sharply above a critical wall shear stress threshold, with reported viability falling to roughly 80% at approximately 2.65 kPa exposure, and nozzle geometry strongly modulates this effect, with cylindrical nozzles causing substantially greater cell damage than conical designs at equivalent flow parameters [18,61]. Current mitigation strategies include keeping extrusion shear stress below this critical threshold, optimizing nozzle geometry, and shear-stress preconditioning of cells prior to printing to upregulate protective stress-response pathways.

Taken together, the convergence of WJ-MSC biology with the technological precision of bioprinting and AI-based quality control outlines a realistic translational pathway for allogeneic cell-based constructs. Pilot clinical indications are likely to include large osteochondral defects of the knee, where off-the-shelf grafts offer clear advantages over autologous procedures in terms of timeliness and scalability [62]. While challenges remain in bioink optimization, immunological safety, and regulatory compliance, the integration of these domains holds the potential to deliver standardized, layered osteochondral grafts that combine biological efficacy with manufacturing reproducibility, ultimately bridging the gap between laboratory innovation and clinical application [63].

### 9.6. Conclusions and Future Perspectives

While Wharton’s jelly MSCs and other stem cell sources already provide a solid foundation for regenerative orthopedic therapies, the convergence of stem cell biology with AI-enabled bioprinting and quality-by-design frameworks is poised to redefine standards of care [51]. This translational pathway may ultimately deliver durable, biologically active, and patient-tailored implants—offering new hope for individuals suffering from degenerative and traumatic musculoskeletal diseases [52].

The application of MSCs—derived from bone marrow, adipose tissue, and Wharton’s jelly—has opened new frontiers in regenerative orthopedics, providing alternatives to traditional arthroplasty and promising outcomes in bone, cartilage, tendon, and intervertebral disc repair [64]. In particular, WJ-MSCs have demonstrated superior proliferative potential, low immunogenicity, and strong immunomodulatory properties, supporting their role as an attractive allogeneic cell source for cartilage and osteochondral regeneration. Current clinical studies confirm their safety and capacity to restore function in degenerative joint conditions, highlighting their translational relevance [53].

At the same time, regenerative medicine is moving toward a new paradigm where AI-assisted bioprinting and multimodal monitoring technologies complement cell therapies [51]. Predictive algorithms trained on rheological and biological datasets are beginning to guide the rational design of bioinks tailored for orthopedic applications, such as osteochondral plugs or tendon-to-bone constructs. Real-time imaging modalities, including OCT and PAI, allow in-process correction of defects and vascular channel verification, while digital twins provide predictive insights into the long-term mechanical performance of grafts under physiological loading [54,55].

For orthopedic surgery, these advances point toward a future in which MSC-based therapies are integrated with AI-driven fabrication platforms, enabling the production of standardized, patient-specific grafts with controlled biomechanical and biological properties [65]. Such constructs could address the critical challenges of osteoarthritis, osteoporosis, osteogenesis imperfecta, and post-traumatic cartilage defects by combining the regenerative potential of stem cells with the precision and reproducibility of autonomous bioprinting. Beyond the manufacturing and imaging considerations discussed above, the broader clinical translation of allogeneic WJ-MSC therapies also depends on addressing donor-to-donor variability in cell potency, the absence of a standardized effective dose across current protocols, the need for systematic post-treatment immune monitoring even for immunoprivileged cell sources, and the current lack of long-term (multi-year) safety data beyond the follow-up windows reported in early-phase trials. Future priority areas therefore include standardized, GMP-validated preparation protocols, adequately powered randomized controlled trials with active comparators, extended long-term follow-up, systematic immunological monitoring, formal cost-effectiveness assessment, and continued alignment with evolving regulatory frameworks for advanced therapy medicinal products.

## Figures and Tables

**Table 1:** Recent clinical trials investigating the therapeutic potential of WJ-MSCs for articular cartilage regeneration and the treatment of knee osteoarthritis.

Clinical Trial ID	Research Center	Study Focus—Key Characteristics	Main Conclusions
NCT03866330	Medical University of Warsaw, Poland	Phase I/II study evaluating intra-articular injection of Wharton’s Jelly–derived MSCs for knee osteoarthritis. Outcomes include KOOS, IKDC, and WOMAC scores assessing pain, function, and joint-specific quality of life.	Trial registered; results have not yet been publicly reported in peer-reviewed literature.
NCT02963727	Investigational clinical study conducted in Jordan	Phase I clinical trial assessing safety and feasibility of intra-articular WJ-MSC therapy in patients with knee osteoarthritis. Primary endpoint focused on safety and tolerability.	Trial registered; no final clinical results have been published in indexed literature to date.
NCT04313894	Erciyes University/Kayseri City Hospital, Turkey	Prospective clinical study evaluating intra-articular WJ-MSC injections for knee osteoarthritis. Outcomes included pain (VAS), function (WOMAC), and MRI-based cartilage assessment.	Demonstrated significant reduction in pain and improvement in functional scores over 12 months, suggesting potential therapeutic benefit.
NCT04520945	Malaysian clinical research consortium	Phase II study investigating UC-WJ-MSC product (“Chondrogen”) for knee osteoarthritis. Clinical outcomes include VAS, WOMAC, IKDC, and KOOS scores.	Trial registered; peer-reviewed outcome data are not yet publicly available.
NCT04863183	Fundación Oftalmológica de Santander—Clínica Carlos Ardila Lulle, Colombia	Phase I/II randomized study comparing intra-articular WJ-MSC therapy (Cellistem-OA) with corticosteroid treatment for knee osteoarthritis. Outcomes include WOMAC, VAS, MRI, and quality-of-life measures.	Early reports indicate a favorable safety profile and greater clinical symptom improvement compared with corticosteroid control.
NCT05160831	Army Medical University (Southwest Hospital), Chongqing, China	Phase I clinical study evaluating repeated intra-articular injections of umbilical cord–derived MSCs isolated from Wharton’s Jelly. Outcomes include safety, WOMAC, VAS, MOCART MRI score, and SF-12.	Demonstrated good safety with no serious adverse events and preliminary evidence of symptomatic and functional improvement.

## Data Availability

The anonymized data will be shared on a special request sent to the authors.
